# Clinical Targets and Attitudes Toward Implementing Digital Health Tools for Remote Measurement in Treatment for Depression: Focus Groups With Patients and Clinicians

**DOI:** 10.2196/38934

**Published:** 2022-08-15

**Authors:** Valeria de Angel, Serena Lewis, Katie M White, Faith Matcham, Matthew Hotopf

**Affiliations:** 1 Department of Psychological Medicine Institute of Psychiatry, Psychology and Neuroscience King's College London London United Kingdom; 2 NIHR Maudsley Biomedical Research Centre South London and Maudsley NHS Foundation Trust London United Kingdom; 3 Department of Psychology University of Bath Bath United Kingdom; 4 School of Psychology, University of Sussex, Falmer East Sussex United Kingdom

**Keywords:** depression, digital health tools, implementation, qualitative, wearable devices, smartphone, passive sensing, sensor data, mobile health, mHealth, mood disorders, digital phenotyping, mobile phone

## Abstract

**Background:**

Remote measurement technologies, such as smartphones and wearable devices, can improve treatment outcomes for depression through enhanced illness characterization and monitoring. However, little is known about digital outcomes that are clinically meaningful to patients and clinicians. Moreover, if these technologies are to be successfully implemented within treatment, stakeholders’ views on the barriers to and facilitators of their implementation in treatment must be considered.

**Objective:**

This study aims to identify clinically meaningful targets for digital health research in depression and explore attitudes toward their implementation in psychological services.

**Methods:**

A grounded theory approach was used on qualitative data from 3 focus groups of patients with a current diagnosis of depression and clinicians with >6 months of experience with delivering psychotherapy (N=22).

**Results:**

Emerging themes on clinical targets fell into the following two main categories: *promoters* and *markers* of change. The former are behaviors that participants engage in to promote mental health, and the latter signal a change in mood. These themes were further subdivided into external changes (changes in behavior) or internal changes (changes in thoughts or feelings) and mapped with potential digital sensors. The following six implementation acceptability themes emerged: technology-related factors, information and data management, emotional support, cognitive support, increased self-awareness, and clinical utility.

**Conclusions:**

The *promoters* versus *markers* of change differentiation have implications for a causal model of digital phenotyping in depression, which this paper presents. Internal versus external subdivisions are helpful in determining which factors are more susceptible to being measured by using active versus passive methods. The implications for implementation within psychotherapy are discussed with regard to treatment effectiveness, service provision, and patient and clinician experience.

## Introduction

The widespread availability of remote measurement technologies (RMTs), such as smartphones and wearables, provides opportunities to assist in the management of patients with long-term health conditions, such as depression. Passive sensing involves the automatic monitoring of behavioral, physiological, and environmental information using multiple digital sensors [1], whereas active sensing requires user input by replying to questionnaires or completing smartphone-based tasks. Used in combination, active and passive monitoring provide ways of capturing continuous, ecologically valid, and high-resolution measures of signs and symptoms related to depression. There are numerous potential uses for such data, including outcome measurements, patient stratification, and clinical decision-making within the treatment.

Cognitive behavioral therapy (CBT) is the first-line treatment for people with mild to moderate depression; however, approximately 50% of patients do not recover from the episode following treatment [2,3]. To date, there are no reliable predictive indicators of treatment outcomes [4]; therefore, a key question is whether RMTs could be used to identify predictors of recovery. For example, RMTs could detect certain behavioral subtypes of depression, which may be more responsive to CBT than antidepressants.

Another application of RMTs is as an outcome measure. Currently, clinical and research outcome measurements rely on infrequent use of symptom scales, which rely on patients remembering and communicating complex mood states, sleep, appetite, and other core symptoms of depression, which is an ability impaired in depression [5]. RMT, by directly measuring key features of depression such as sleep and activity, may provide more valid indicators of core depression phenomenology and therefore provide better measures of treatment outcomes.

A further application of RMTs would be to provide information to clinicians and patients, which could enhance the delivery of care. For example, the impact of interventions such as sleep hygiene or behavioral activation could be observed by patients and clinicians using RMT, which would provide a more direct and continuous indicator of change in targeted behaviors than when relying on diaries or conventional outcome measures.

The measure of behaviors of interest in depression has taken a *bottom-up* or data-driven approach to infer clinical states from digital sensors; digital markers of behavior have adjusted to existing sensors rather than the other way around [6]. These approaches are helpful in identifying potential digital biomarkers of disease; however, they are not always clinically meaningful. To generate clinical targets relevant to a patient’s needs, their views on what illness or improvement looks like for them should be included. Clinical outcomes in digital health research are based on mainstream diagnostic scale items; however, even established psychometrics are criticized for overlooking outcome domains that are important to patients [7]. Therefore, patient-centered approaches to digital mental health are needed.

User acceptability is at the core of technology adoption [8], and thus, the patient and clinician acceptability of RMTs is crucial for successful implementation within the treatment. Although RMTs have shown adequate levels of acceptability [9], few studies have explored in-depth views on patient experience [6], and those conducted in a clinical setting are fewer still [10]. This is problematic, given that treatment-seeking populations have the potential added acceptability considerations of increased burden of symptom severity, low cognitive and emotional resources [11], and ethical concerns surrounding personal data sharing [12].

In addition to barriers, identifying and harnessing facilitators of RMT use during treatment may further motivate their use within services. Simblett et al [11] found that patients felt that RMTs provided opportunities to connect with peers and brought about a sense of control and understanding of their condition. However, previous studies have mostly considered participants who were already using health tools as part of a research study [13-15]. Although the experiences of current users are undeniably helpful, understanding pre-use attitudes toward new tools, including barriers to and facilitators of their adoption, is central to their uptake and implementation in health care services.

Therefore, the objectives of this study were 2-fold. Using qualitative methods, we first aimed to understand what outcomes are important for clinicians and patients as they improve with treatment to create clinically meaningful targets for digital health research. Second, we aimed to explore patient and clinician attitudes toward the use of RMTs and identify any perceived barriers to and facilitators of using these methods during psychological treatments for depression.

## Methods

### Design

This was a qualitative study with a focus group design. A thematic analysis was used to identify overarching themes within the participants’ attitudes and experiences. The topic guide was developed based on the research goals, where the 2 main aims were allocated to approximately half of the session each.

### Participants

We recruited a total of 22 participants, of whom 16 (73%) were current or recent users of the Improving Access to Psychological Therapies (IAPT) Talking Therapy program, a psychotherapy delivery service provided by the United Kingdom’s National Health Service (NHS). A separate group of clinicians within the IAPT program were also recruited (6/22, 27%). They either delivered CBT or were part of the care team for patients undergoing CBT for depression and anxiety.

Clinicians with at least 6 months of experience in their roles were recruited via email through their services. Patients were recruited through advertisements in mental health service waiting rooms and advertisements circulated by the staff. Patients were eligible if they were aged ≥18 years and had received at least three sessions of IAPT-delivered CBT for a depressive disorder in the past year.

### Ethics Approval

The study was reviewed by the London Bridge Research Ethics Committee, and approval from the Health Research Authority was obtained (reference 19/LO/0662).

### Patient and Public Involvement

This research was reviewed by a team with experience with mental health problems and their caregivers who were specially trained to advise on research proposals and documentation through the Feasibility and Acceptability Support Team for Researchers—a free, confidential service in England provided by the National Institute for Health Research Maudsley Biomedical Research Centre via King’s College London, South London, and Maudsley NHS Foundation Trust. The reviewed documentation comprised all participant-facing documents, excluding the interview schedule.

### Procedure

A total of 3 separate focus groups led by 2 researchers were conducted in August 2019 at King’s College London, 2 for patients and 1 for clinicians. All participants provided written informed consent to participate in this study. Patient sessions ran for 2 hours and clinician groups for 1 hour. The participants were compensated £25 (US $32.50) for participating in the study.

Baseline demographic data, including age, gender, ethnicity, and the type of treatment received or administered, were recorded. During the focus groups, participants were prompted with a series of prespecified questions to explore the outcomes that people value in relation to their mental health (eg, improved sleep, increased socialization, and completion of daily chores). In addition, they were asked about their attitudes toward using smartphones and wearable technology during psychological treatment (see supplementary note 1 for the topic guides in Multimedia Appendix 1). Sessions were audio recorded and transcribed verbatim (excluding filler words, such as “erm” or “um”). The same 2 researchers—a PhD candidate and a postdoctoral health psychologist—facilitated all the sessions.

### Data Analysis

Transcriptions were independently coded and analyzed by 2 researchers using the NVivo software (version 12; QSR International). To improve the breadth of perspectives and reduce researcher bias, coding was replicated by a qualitative researcher who was not present in the focus groups.

Following Braun and Clarke [16], an inductive, thematic analysis approach was followed, in which the data drive the generation of themes rather than a previous theoretical basis. The researchers read the transcripts to identify emerging themes. Recurring topics were grouped under the same code until the general patterns of themes were identified. The researchers then met to discuss and consolidate themes, generating a new list of codes, which was used for a further round of coding.

## Results

### Sample Demographics

There were a total of 22 participants. The main demographic characteristics of the sample are presented in Table 1. All patients had a diagnosis of depression, and 63% (10/16) reported comorbidities. Half of the participants (8/16, 50%) reported having comorbid anxiety disorders, including generalized anxiety disorder and panic disorder. The results were separated into two main sections: (1) relevant clinical outcomes and (2) implementation barriers and facilitators.

**Table 1:** Demographic characteristics of participants in the clinician focus group and both patient focus groups (N=22).

Characteristics		Clinicians (n=6)	Patients		Total (N=22)
			Group 1 (n=9)	Group 2 (n=7)	
Age (years), mean (SD)		36.7 (9.3)	47.9 (15.7)	47.7 (11.6)	44.6 (13.3)
**Gender, n (%)**					
	Women	5 (83)	6 (67)	7 (100)	18 (82)
	Men	1 (17)	3 (33)	—^a^	4 (18)
**Ethnicity, n (%)**					
	Asian or Asian British	1 (16)	—	1 (14)	2 (9)
	Black African or Caribbean, or Black British	1 (16)	3 (33)	—	4 (18)
	White British	3 (50)	4 (44)	4 (57)	11 (50)
	White other	—	1 (11)	1 (14)	2 (9)
	Mixed or multiple ethnic groups	1 (16)	1 (11)	1 (14)	3 (14)
Comorbid anxiety diagnosis, n (%)		N/A^b^	4 (44)	4 (57)	8 (50)^c^
Physical health condition, n (%)		N/A	2 (22)	1 (14)	3 (19)^c^

^a^Not available (no participants with these characteristics).

^b^N/A: not applicable (diagnosis information not collected for the clinician group).

^c^Patient data only; total: N=16.

### Clinical Outcomes

#### Overview

**Figure 1:**
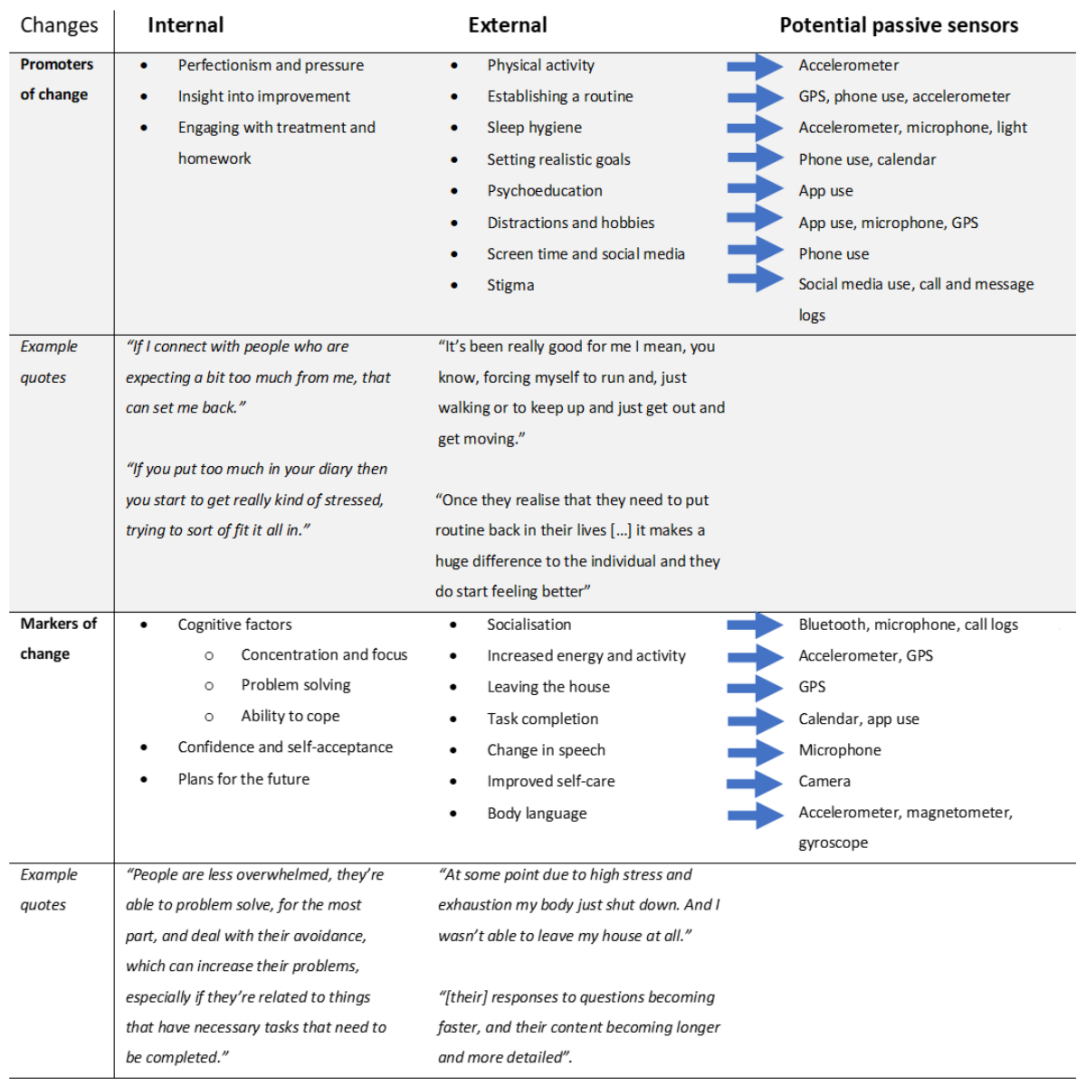
Internal and external markers and promoters of change and their corresponding remote measurement technology sensors through which they could be measured.

The first section of the focus group discussions revolved around identifying which behavioral changes signaled an improvement in, or worsening of, mood to our participants. Two main themes were identified: behaviors that people engage in, which have an impact on mood, named here as *promoters of change*, and behaviors that manifest because of a change in mood, termed as *markers of change*. These themes were further subdivided into either external changes (referring to changes in their behaviors and could, in principle, be measured objectively) or internal changes (referring to changes in thoughts or feelings rather than actions). Figure 1 summarizes the markers and promoters of change discussed in the groups with example quotes.

#### Promoters of Change

##### Internal

Three main subthemes emerged as internal processes that could help promote or hinder improvements in people’s moods. First, participants found that feeling pressure from themselves or striving for perfectionism to be *better* or *do more* would hinder their improvement. Second, they felt that having insights into their illness and awareness of progress was gratifying. Third, experiencing an improvement in mood led some people to gradually disengage with treatment “instead of doing the work again so you do get complacent” (P07) by leaving homework incomplete or stopping the medication, which, in consequence, worsened mental health.

##### External

A prevalent subtheme for all focus groups as a promoter of mental well-being was staying active, including both vigorous exercise and milder physical activity. This was followed by establishing a routine, which both clinicians and patients found to be central to experiencing improvement. Striving to create better sleep habits to improve mental health and setting realistic goals in the short term gave people a sense of accomplishment. However, unrealistic targets ran the risk of bringing discouragement and worsening the mood if left incomplete.

Psychoeducation, including signing up for and engaging with mental health courses, was perceived as helpful, as was completing the homework assigned during therapy. In addition, participants found value in finding suitable distractions such as comedy shows, music, and pursuing hobbies. Notably, only clinicians discussed social media and screen time as having a potential impact on mental health.

Societal stigma or how other people reacted to participants’ mental health difficulties was discussed only by patients as something that affected their mental state but recognized that they had no control over it.

#### Markers of Change

##### Internal

As people experienced improvement, they reported a general feeling of being better able to cope, feeling less overwhelmed with daily difficulties, and more motivated to go about their daily lives. A participant summarized it as “I find I’m able to run my day better” (P13). Participants also reported an increase in confidence, self-acceptance, and making more plans for the future, as their symptoms improved.

##### External

The single most mentioned theme was socialization and how, as mood improved, they “began to reach out to friends again, to reconnect with them socially” (P07). Going out more often and meeting an increased number of people were mentioned.

Lack of energy, both physically and mentally, was discussed as one of the more noticeable things, particularly with reference to being able to leave the house as a combination of both physical and mental motivation. Clinicians pointed out that “people become more active, don’t they, as we go through therapy” (C02).

Successful completion of daily tasks and other small daily achievements was highlighted, which clinicians believed represented an improvement in cognitive function. Specifically, people noticed they were able to “get things off my to-do list rather than just postponing them” (P01).

Clinicians reported noticing changes in a patient’s speech; however, one of the clinicians pointed out that speech change patterns may be different in anxiety, which, in contrast to those with depression, may be faster to answer when symptoms are more severe. Changes in self-care habits were also reported by both clinicians and patients.

The patients expressed the importance of body language and how others could discern their mood from their physical appearance and facial expressions.

### Implementation Barriers and Facilitators

**Figure 2:**
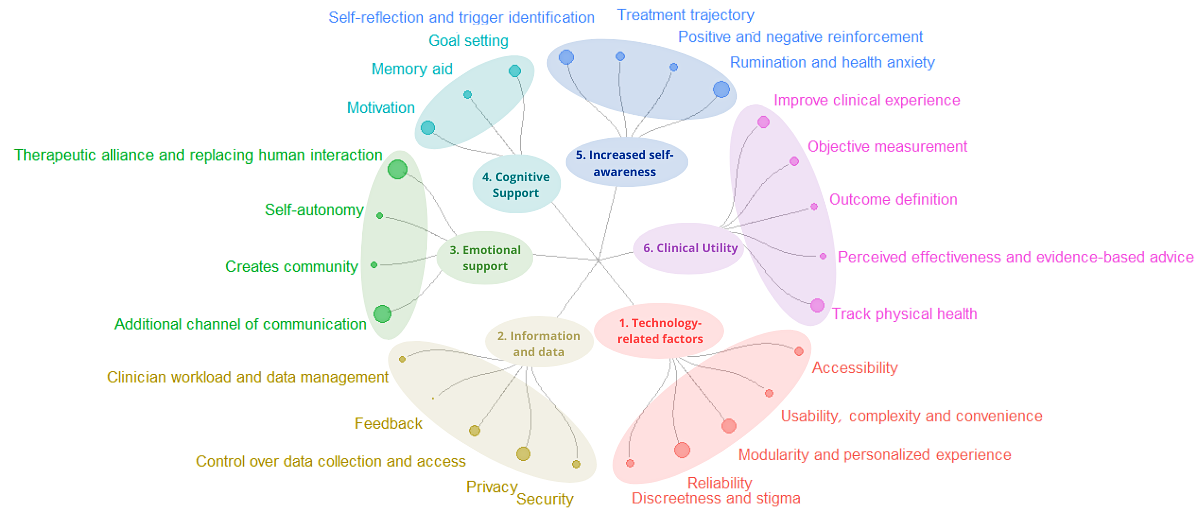
A breakdown of the six themes and subsequent subthemes emerging from the data. End nodes correspond to the number of codes related to each subtheme; the larger the node, the more instances of coding for that subtheme.

The second part of the study focused on how RMTs, with the potential to measure the aforementioned symptoms, were perceived by patients and clinicians. A total of 6 overarching themes and 26 subthemes were identified (Figure 2), and quotes for each subtheme are presented in Table S1 in Multimedia Appendix 1.

#### Technology-Related Factors

Under the first theme of technology-related factors, we identified the subthemes of accessibility, usability and convenience, personalization of experience and modularity, reliability, and discreetness or stigma. Most participants were concerned about the price of the technology and the surrounding infrastructure, including access to reliable Wi-Fi. Participants also agreed that technology and software would need to be simple to use and convenient; for example, requiring regular charging or software updates that took time and technological literacy would affect their engagement. Participants valued the option to personalize their experience and adapt the software to their needs by deciding what to measure and manipulating the screen display. Clinicians agreed that modularity within the software would also be useful for them.

Concerns arose regarding the reliability of the technology, especially devices with physiological readings, and how making health decisions based on unreliable sensors could potentially be harmful. Discretion was also important, as wearable devices should not identify people with psychiatric disorders. Participants preferred mobile phone apps to wearable devices; however, commercially available fitness devices or smartwatches would avoid these concerns.

#### Information and Data

Data and information management emerged among the three focus groups and revolved around five main topics: security, privacy, control over data collection, feedback, and clinician data management and workload. Compared with patients, clinicians verbalized more data considerations.

Within the subtheme of security, participants were mainly concerned about sensitive information being leaked or picked up through digital sensors and going out to third parties and private corporations. Privacy concerns were related to who could gain access to the data and what choice patients would have over their access. In addition, patients wanted control over data collection and the option to engage with this technology or opt out rather than being a prerequisite for treatment. Therefore, many participants preferred the idea of a wearable device over a smartphone app for health or research purposes, as having a separate device gave participants a sense of awareness and control over when data collection took place.

Patients generally wished to review their progress, and thus, receiving feedback from RMTs would be valuable but should be tailored according to personal preferences and in a way that promotes positive reinforcement.

For clinicians, themes emerged related to managing big data, training, and clinical workloads. They were concerned about receiving adequate training, which would affect the effectiveness of the tool and their overall workload, and discussed the necessity of software to help sift through large amounts of information to generate actionable insights. Clinicians raised further concerns about whether RMT data would be used to evaluate risk and whether this would add to their workload.

#### Emotional Support

Attitudes toward whether RMTs could provide an additional source of emotional support during treatment went in 2 opposite directions. On the positive end of the spectrum, participants believed that digital tools could complement human interaction by creating another channel of communication, which was thought to help with in-treatment discussions. Both clinicians and patients discussed how RMTs could accommodate the provision of lower-intensity extra support after treatment, for example, by having posttreatment check-ins. This technology was also thought to provide an opportunity to create a community by sharing achievements and involving others in their goals, thus providing an additional source of support.

Conversely, 2 further subthemes revolved around the fear of overreliance on technology and a decline in human interaction. Clinicians believed that relying on a wearable device during treatment could affect relapse rates if patients became reliant on these tools and they were then taken away after treatment. Patients echoed this sentiment and added that this overreliance may detract from a feeling of autonomy.

Any replacement of human interactions was almost universally considered detrimental to the therapeutic alliance. Participants believed that no longer feeling accountable to a therapist would detract from their motivation to engage with treatment or homework.

#### Cognitive Support

The positive motivational impact of automated messages or notifications being delivered to people via smartphones was discussed in all groups. Participants thought positively of RMTs as tools for memory aids in several ways, including helping manage practicalities such as taking medication and keeping track of what improves their mood. Clinicians believed that using RMTs as memory aids could improve adherence to homework or medication. Another way in which it was perceived as a complement to treatment was through planning and goal setting.

#### Increased Self-awareness

Subthemes within self-awareness revolved around having a reflexive tool to identify triggers and boosters of well-being, assess treatment trajectory, positive or negative reinforcements, and the potential to worsen rumination and health anxiety. Keeping a continuous log of these reflections was thought to help identify changes in symptom trajectories in an ecological context, such that it could provide guidance on what improves people’s mood and identify negative triggers.

Clinicians discussed the benefits of potentially identifying treatment trajectories, such that they may adjust strategies depending on changes in behavior. A subtheme of positive reinforcement emerged in the groups, in which people believed that being shown progress could prove motivating. Others advised caution, as being presented with failure to achieve goals could lead to feelings of self-defeat. Being forewarned of a possible dip in mood gave some people a sense of foreboding, which they felt could create a self-fulfilling prophecy.

A prevalent concern was whether monitoring health would lead to increased anxiety as people obsess over data, which could, in turn, worsen existing symptoms such as lack of sleep. Indeed, some people disliked the idea of having a regular request to reflect on their mental state and, therefore, constant reminders of their ill mental health. Clinicians highlighted the risks to those with health anxiety if they were exposed to constant health monitoring and feedback.

#### Clinical Utility

The theme of clinical utility emerged in relation to how, and to what extent, digital tools could complement treatment. The identified subthemes were related to improving the clinical experience, objective symptom measurement, outcome definitions, treatment effectiveness or concerns over the evidence base, and targeting physical health.

RMTs were thought to improve the clinical experience by replacing questionnaires, offering an electronic alternative to paper, and gamifying data collection. Both patient groups expressed a strong dislike for repeated symptom measurement questionnaires, and automatic data collection was considered a pleasing alternative. For aspects of therapy that cannot be fully automated, such as homework, participants still expressed a stronger disposition to engage with electronic methods.

Clinicians discussed the utility of more objective behavioral or physiological symptom measures that could help contextualize a patient’s symptoms. At the same time, they warned that objective benchmarks for outcome definitions would vary drastically across patients, such that symptom improvement may manifest with opposite digital signatures across different conditions.

Both patient groups discussed the issue of trust and whether technology was as effective as human-based treatments in the context of treatment. It was important for all groups that any recommendations based on digital tool readings be evidence based or accredited by the NHS to ensure their reliability.

An additional discussed benefit of using these devices in a clinical population was that they encourage improvement in physical health and sleep, which are items reportedly targeted in treatment.

## Discussion

We aimed to understand what improvement-related outcomes are important for clinicians and patients, as well as the attitudes toward the use of RMTs within psychological treatment. The main purpose of this study was to generate clinically meaningful targets for RMT research and help in their implementation in research and clinical practice.

### Clinical Outcomes

Participants talked about the behavioral, cognitive, and emotional changes experienced alongside mood fluctuations. Emerging themes were categorized as either markers or promoters of change to differentiate how participants viewed these fluctuations. Markers of change seemed to be referred to as consequences of improvement in mental state, whereas promoters were viewed as thoughts or behaviors that were perceived as affecting changes in mental state.

This differentiation has implications for the causal model of digital phenotyping in depression. Undoubtedly, patient and clinician perspectives do not necessarily reflect the reality of the time order relationship of these symptoms, which is a necessary characteristic of causal inference. However, this model can serve as a useful framework to contextualize the analysis of relationships between digital features. This is especially relevant as, with few exceptions [17], much of the existing work on digital mental health is based on correlations [18], and determining causality is necessary to extract actionable insights, especially as they relate to treatment [19].

**Figure 3:**
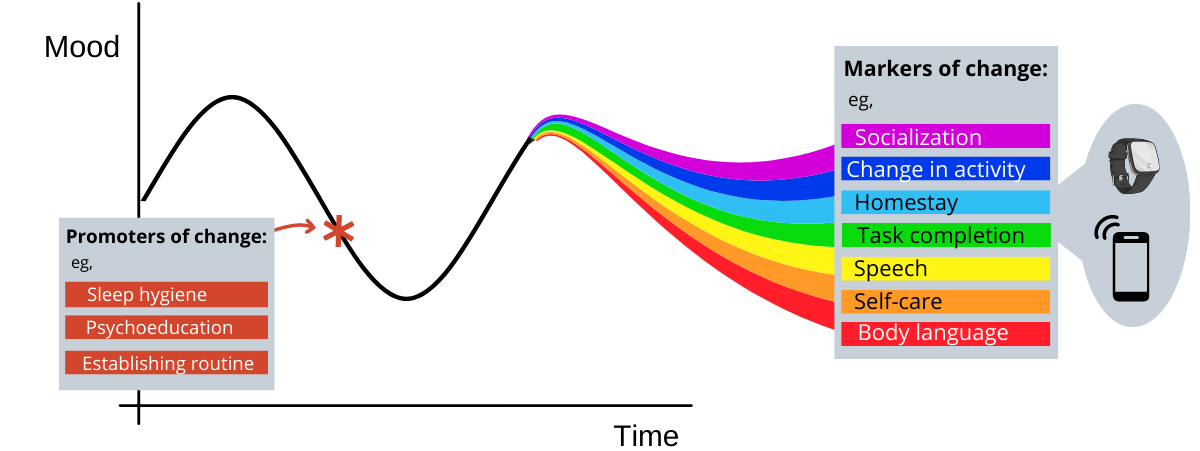
Model depiction of promoters and markers of change. As mood fluctuates, so do the markers of change, such as socialization, homestay, or speech, each represented by a colored line. These vary with mood and can be used in combination to assess the current mental state. Promoters of change, such as routine, sleep hygiene, and psychoeducation, can be viewed as clinical targets, which can be actioned at a time of downward mood trend (as depicted by the red asterisk) and promote improved mental health.

In this model, depicted in Figure 3, markers of change could be used as a proxy for changes in mental state—the *effect* in a causal effect model—and, therefore, primary targets for the remote monitoring of symptoms. However, promoters of change may be unrelated to the *current* mental state but be actionable targets for treatment and predictors of mood fluctuations.

The subdivisions of internal versus external factors could be helpful in determining which factors are more susceptible to measurement with active versus passive methods. GPS and accelerometer sensors have been widely used for this purpose, with promising results [20,21]. Other factors, such as body language (gait and posture), maintenance of a routine, self-care, and task completion, have a very scarce literature base if any (eg, gait [22] and task completion [23]), likely because of the difficulty in operationalizing them via remote sensing.

Despite the advantage of minimal engagement required for passive sensing, data from active apps such as smartphone calendars can provide low-burden information on items such as workload, leisure activities, and completed daily tasks. For example, Wahle et al [23] included calendar events in prediction algorithms to distinguish people with depression from nondepressed people. However, it is likely that a multidomain approach to data collection would be preferable to capture the complex nature of the behaviors of interest.

Questions have arisen regarding the accuracy and face validity of digital sensors that genuinely detect the behavior they claim to measure [24]. However, sensor data have been widely found to have adequate construct validity in that they can predict self-assessed moods [18]. This means that although caution is advised when inferring real behavior from digital features, they may serve as helpful signals for depression.

### Implementation Barriers and Facilitators

#### Overview

The implementation of digital health tools in health care services requires insights into barriers and facilitators from stakeholders. In the second section of our focus group discussions, we identified six main themes: technology-related factors, information and data, emotional support, cognitive support, increased self-awareness, and clinical utility. The implications and impact of these themes on implementation within treatment with regard to treatment effectiveness, service provision, and patient and clinician experiences are discussed in the following sections.

#### Implications for Treatment Effectiveness

One of the necessary drivers of implementation is that these tools are perceived as improving treatment outcomes. The perception that RMTs could provide better communication and increased self-awareness are 2 routes through which this could be achieved. The third route is through supporting homework [25]. Being able to follow patient trajectories more closely would aid the development of prediction models for recovery and relapse [15] and help clinicians make prompt and better-informed decisions [26]. In addition, integrating a treatment platform where patient-clinician communication is enhanced with physical health tracking, psychoeducation, and memory and motivational aids that are specifically tailored to patient symptoms and treatment schedules could boost effectiveness and move toward a more holistic and personalized approach to treatment.

The patient-clinician therapeutic relationship was one of the most prominent themes, where concerns that it may replace human-delivered treatment can feel dismissive and indeed hinder treatment effectiveness, despite finding value in the extra low-intensity support technology provided. Despite the dearth of research on the effect of digital health on therapeutic relationships [27], a recent narrative review suggests that an alliance within digital mental health can be cultivated, although it may take a different form [28]. Given that a therapeutic alliance is a highly predictive factor for treatment response [28], such concerns should be carefully considered in implementation strategies [29].

Importantly, RMT capabilities in terms of feedback schedules need to be tailored to individual needs so as not to exacerbate symptoms. Generally, feedback was appreciated; however, close monitoring was strongly discouraged for those with health anxiety. Indeed, case studies from the physical health field have found that wearables worsen mental health in such cases [30].

#### Impact on Service Provision

Service provision could be negatively affected by the broadening of the digital divide—the gap between those who benefit from the digital age and those who do not. This study found that technology-related factors such as accessibility, usability, and complexity could affect the breadth of service provision by increasing the digital divide in three main ways: those with lower technology experience and literacy are less likely to benefit from technologies with more complex designs [31], people with severe depression are more likely to be unemployed and therefore have lower purchasing power for devices or stable internet [32], and reduced cognitive abilities could affect the capacity to effectively interact with the devices.

Despite research linking remote monitoring to increased overall access to health care [33] and an overall increase in the capacity for service provision, inequalities may still appear if those with less access, experience, or capacity are less likely to benefit from this technology.

#### Patient Experience

The main potential for improvement in patient experience is by replacing recurring symptom questionnaires, which were almost unanimously disliked by patients, who disliked the repetition and negative phrasing of their symptoms. This is supported by previous research showing that patients with depression would value measures of wellness and illness [34]. Digital active data collection methods could allow for different ways of capturing symptoms, such as visual or animated scales. Their use could also be reduced altogether by incorporating passive sensing.

Additional patient concerns revolved around data, specifically around having agency over data collection and access, a finding replicated elsewhere [11]. Therefore, patient-clinician discussions on which behaviors to monitor, and why, could improve attitudes toward RMT use in treatment. Privacy concerns could be reduced for some people if certain features, such as location-based data, were not deemed relevant to a patient’s treatment and, therefore, not recorded.

#### Clinician Experience

A key facilitator for clinicians was the utility of having objective behavioral measures to contextualize symptoms. They believed that this could be used to motivate and engage patients in their care. However, a major factor that could affect clinician buy-in was related to data management and interoperability, a concern also found in primary care settings [35]. Electronic medical record systems are already a source of excess data inputting for clinicians. Adding active and passive data generated from digital health tools is unlikely to improve care and may well overburden clinicians unless they are provided with adequate tools and training to manage and interpret data in actionable ways. Much of the research on acceptability revolves around patients; however, clinicians are end users who have been shown to express varying attitudes toward the implementation of new technologies, attitudes that can be improved through exposure to technology and training [36].

### Strengths and Limitations

This study included clinician views, a key stakeholder with a strong influence on implementation success and whose practice we aim to influence. The inclusion of 3 separate focus groups allowed for different dynamics and a wider range of experiences to be captured in this study.

Some limitations include the fact that despite the importance of capturing attitudes before implementation, the barriers and facilitators reported here may vary after the experience of sustained RMT use. This study relied on a small sample of participants with treatment experience from a single health service, meaning that these results may not be broadly generalizable to other settings.

In addition, the participants’ previous experiences with technologies were not included in the analysis.

There may also be confusion surrounding the term *RMTs* and what they can be used for; thus, a dichotomy may arise between the use for self-management, which patients are more likely to envision, or the use for prediction in clinical care. The latter is less understood and thus might have affected the participants’ responses.

### Future Research

Future longitudinal studies could include active data that capture the emotional and psychological factors presented in this study and could generate passive data features that closely match the behavioral markers reported here. Studies examining the severity of depression and device engagement are already underway [37]. Applying such methods to patients currently in psychotherapy could provide insights into how real engagement and device use vary within mental health services. Finally, the study by Torous et al [38] sheds light on the importance of supporting working alliances in digital health platforms; therefore, similar studies examining remote measurement and digital therapeutics are necessary.

### Conclusions

Digital tools bring new methods of data collection and new outcomes; therefore, there is a need for a re-evaluation of the clinical targets that are considered important. Therefore, advice on what type of information should be measured and analyzed, as well as attitudes toward their use from different stakeholders, is central. This study used a qualitative approach to identify clinical targets that are important to patients and clinicians and developed a framework for determining which factors may be more susceptible to be measured using active or passive methods. We also identified 6 main themes surrounding attitudes toward RMTs, which could drive implementation efforts in health care settings.

## Appendix

Multimedia Appendix 1 Topic guide and patient and clinician quotes for themes and subthemes.

## Abbreviations

CBT: cognitive behavioral therapy
IAPT: Improving Access to Psychological Therapies
NHS: National Health Service
RMT: remote measurement technology
