# Supplementary material for: Clinical Targets and Attitudes Toward Implementing Digital Health Tools for Remote Measurement in Treatment for Depression: Focus Groups With Patients and Clinicians
Source: JMIR Ment Health. 2022 Aug 15;9(8):e38934. doi: 10.2196/38934 (PMC9425163; doi:10.2196/38934)
Supplement: Multimedia Appendix 1 [file mental_v9i8e38934_app1.docx]

# **MULTIMEDIA APPENDIX FOR**

**Clinical Targets and Attitudes Toward Implementing Digital Health Tools for Remote Measurement in Treatment for Depression: Focus Groups With Patients and Clinicians**

*Valeria De Angel* ^1,2^, Serena Lewis ^1,3^, Katie White ^1^, Faith Matcham ^1^, Matthew Hotopf ^1,2^*

1. Institute of Psychiatry, Psychology and Neuroscience, King's College London, London, UK
2. NIHR Maudsley Biomedical Research Centre, South London and Maudsley NHS Foundation Trust, London, UK
3. Department of Psychology, University of Bath, Bath, UK

* Corresponding Author contact details:

E3.08, 3rd floor East Wing, Institute of Psychiatry, Psychology and Neuroscience

16 De Crespigny Park, London, SE5 8AF

E: [valeria.de_angel@kcl.ac.uk](mailto:valeriadeangel@gmail.com%09)

## **Supplementary Note 1: Topic guides for Patients and Clinicians**

**Focus group topic guide – Patients**

**Introduction**

- Researchers to introduce themselves and the project
- Introduce audio recording equipment, how long the session will last and breaks
- Explain confidentiality (including use of quotes) and agree ground rules
- Explain consent procedure: go through information sheet and sign written consent forms
- **Ice breaker:** Introduce yourself and say how you travelled in today.

**Main discussion**

1. Aim: to understand what is important to you about the changes you experience when you improve in the context of undergoing treatment for depression; to be able to identify treatment targets of importance
   - Is measuring changes during treatment important to you?
   - What else is important to you in terms of your (physical and mental) health and well-being? Why?
     - Prompts: Physical and mental health; recovery; lifestyle; social relationships
   - What sort of changes do you notice when you start feeling a better while receiving treatment?
     - Prompts: things do you notice you do more (or less) of
   - Do you regularly measure your health? How? Do you share this with anyone?

*NOTE TO MODERATOR: Explain that project is interested in seeing how wearable and mobile technology can be used to predict some of the changes that people experience during treatment that have already been discussed.*

1. Aim: to discuss use of wearable and mobile technology and its association with health and well-being; what are the barriers and facilitators to this? *MODERATOR: Provide examples of wearable technology, e.g. heart rate monitor, pedometer to measure steps.*
   - Who has used wearable or mobile technology to measure health and well-being? Or knows of someone else who has? What was it for? If not, why not?
     - Probe: which devices (e.g. smartphones or watches)?; measuring symptoms associated with condition?
     - Prompt activity monitoring, alerts and reminders etc.
   - How did you feel about using wearable or mobile technology?
     - Probe: barriers and facilitators, e.g. what encouraged you to use it? And keep using it? What got in the way?
   - How would you feel about using wearable or mobile technology to predict changes in your condition?
     - Probe: motivators and barriers/concerns
2. Would you like to add anything else?

**Close**

- Explain how data will be used
- Remind participants about confidentiality
- Explain the next steps in the RAPID project.

**Focus group topic guide – Clinicians**

**Introduction**

- Researchers to introduce themselves and the project
- Introduce audio recording equipment, how long the session will last and breaks
- Explain confidentiality (including use of quotes) and agree ground rules
- Explain consent procedure: go through information sheet and sign written consent forms,
- **Remind participants although it is likely some of them will have experienced depression or have cared for someone with depression, that they are here in their role as clinicians, and this is the view we would be most interested in at this time.**
- **Ice breaker**: Introduce yourself and say how long you have been doing CBT for people with depression for.

**Main discussion**

- - - 1. Aim: to understand what is important about the changes people with depression experience when they improve in the context of undergoing treatment for depression; to be able to identify treatment targets of importance.
- Is measuring changes during treatment important?
- What else do you feel is important to patients in terms of their (physical and mental) health and well-being? Why?
  - Prompts: Physical and mental health; recovery; lifestyle; social relationships
- What sort of changes do you notice when they start feeling a little bit better while receiving treatment?
  - Prompts: things you notice they do more (or less) of

*NOTE TO MODERATOR: Explain that project is interested in seeing how wearable and mobile technology can be used to predict some of the changes that people experience during treatment that have already been discussed.*

- - - 1. Aim: to discuss use of wearable and mobile technology and its association with health and well-being; what are the barriers and facilitators to this? *MODERATOR: Provide examples of wearable technology, e.g. heart rate monitor, pedometer to measure steps.*
- Who has used wearable or mobile technology to measure health and well-being? Or knows of someone else who has? What was it for? If not, why not?
  - - Probe: which devices (e.g. smartphones or watches)?; measuring symptoms associated with condition?
    - Prompt activity monitoring, alerts and reminders etc.
- How did you think people with depression might feel about using wearable or mobile technology before and during treatment?
  - - Probe: barriers and facilitators, e.g. what do you think would encourage them to use it? And keep using it? What would get in the way?
- How do you think they would feel about using wearable or mobile technology before and during treatment to predict changes in their condition?
  - - Probe: motivators and barriers/concerns
      1. Would you like to add anything else?

**Close**

- Explain how data will be used
- Remind participants about confidentiality
- Explain the next steps in the RAPID project.

## **Supplementary Table 1: Quotes from patients and clinicians.**

**Table 1.** Patient and clinician quotes for themes and subthemes.

| Theme and subtheme | | Patient quote | Clinician quote |  |
| --- | --- | --- | --- | --- |
| **Technology-related factors** | | | | |
|  | Accessibility | “my eyesight is getting older and if it’s got tiny, tiny print on it and then you can’t read it.” | “Challenges would be for people who, just because of socioeconomic issues, don’t really have access to certain technology.” |  |
|  | Usability (complexity and convenience) | “I don’t think- If we have to recharge it- remember to do that every day.” | “I think a key thing is making it as simple as possible as well as the technology because then people are more likely to engage with it” |  |
|  | Modularity (personalized experience/use) | “If I can tailor it a little bit more, I might be more motivated to use it on a regular basis because for me, it’s important” | “I think it would be helpful to have a key routine number of different items, whether it’s steps, whether it’s heart rate or whatever, that are standard, and then a few extra ones that could be added on as necessary depending on who you’re working with, to make it more fitting with whatever their presentation is.” |  |
|  | Reliability | “[making the analogy to blood pressure] if such an app is not working well, it can bring even more damage. Example, if I’d sent it without checking to my parents, and they were ‘okay so, oh my blood pressure is too high, that means I need to take the meds’ that would kill my parents.” | “all available apps for health tracking should be authorised [...] they should be checked by health professionals because if such an app is not working well, it can bring even more damage” |  |
|  | Discreetness+stigma | “I think an app is better than something—You don’t want to flag it up to everyone that you’re having treatment or something really.” | “I think it would really help with the stigma of being in therapy as well, because yeah if it’s attached to a mobile or a watch that most people wear, no one’s going to think about it twice. And it’s a nice way people can engage in therapy without being worried that people are going to find out.” |  |
| **Information and data** | | | | |
|  | Security | “I’ve often thought if I’m on the phone giving my bank details, that [device] thing could be listening to that. I start thinking, ‘Has somebody got my bank details?’ Because it kind of listens to everything you say.” | —^a^ |  |
|  | Privacy | “I find the idea of something that tracks my activity terrifying and not something that I’d want to willingly sign up to. A world that involves more surveillance and data use- like that makes me feel sick.” | “I think it’s about having a conversation with the client at the beginning about boundaries really, and once it’s clearer, how the information will be shared and can be shared, then you can—Normally put in those boundaries and then you can understand those concerns.” |  |
|  | Control over data collection+access | “I’d feel better about it not going without my knowledge to doctors” | “Maybe there’s also an agreement there about how often the information will be checked and can be checked either by clinicians or the client.” |  |
|  | Feedback | “[clinicians] should be sharing it with us so that we can see that actually even though if we don’t feel we’re improving, the metrics are showing that there is improvement. The trajectory is in the right direction, even if the blips are going up and down, all over the place.” | “to have a way to just, very briefly, summarise that data in progress weekly, can actually be really nice. And you can dictate when to review it, what to tweak, what to change.” |  |
|  | Clinician workload+data management | — | “being able to have a really simple, easy way to compare the progress throughout the weeks of treatment. So you would obviously be collecting a huge amount of data but if there was a way that we could somehow get, ‘Okay, you did an average of X amount of steps in week one, and your average sleep was X amount with waking up X amount of times.’”  “as long as we’ve had adequate training. [...] And it’s not just having the training, it’s then having the time to think about that afterwards and incorporate it into your practice which would require a corresponding decrease in clinical word.” |  |
| **Emotional support** | | | | |
|  | Additional channel of communication | — | “And I think that might be helpful in facilitating discussion. Make it easier for people to talk about. And I think that would be the case with this type of technology wouldn’t it. It bypasses any sort of (worry) that as a patient they’ll have in telling you about things. Whether that’s that they didn’t do their homework...” |  |
|  | Creates community | “-an unexpected benefit is my family—my children—they will ask me how many steps do I still need to get, and they will go for a walk around the block with me in the evening.” | — |  |
|  | Self-autonomy | “Sometimes you have to do something on your own. You can’t just keep on getting monitored.” | “if there’s constant messages between sessions like, ‘Remember to do this. Remember to do that.’ If this watch is taken away at the end of therapy, they will kind of relapse quite severely I think because they’re not learning to be self-autonomous.” |  |
|  | Therapeutic alliance/replace human interaction | “we already feel isolated enough. It must never replace human interaction because otherwise I don’t think any of us would get better.” | — |  |
| **Cognitive support** | | | | |
|  | Motivation | “It might help me keep on track and set up good practice for the future and maintaining positive—good health—healthy wellbeing” | “Or just encourage them—nice messages throughout the day which is sometimes what people want to do for themselves anyway.” |  |
|  | Memory aid | “With those smart watches, they’ve got another useful setting. I’ve got a lot of problems with concentration and I don’t control time at all. I don’t have a clue how much is five minutes and how much is one hour because my mind is hurting [...] so I kept reminders for my medication” | “so much of the homework or the therapy is based on scheduling so sometimes you can give them reminders or they can do it some way through the app.” |  |
|  | Goal setting | “you could make some really good goals. Kind of like, oh if you want to do more exercise, I’m gonna walk like 3000 steps a day and be able to monitor that every day” | “you can even compare what [patients] have planned or what they have actually done” |  |
| **Increased self-awareness** | | | | |
|  | Tool for reflection/identify triggers | “for me, it’s important to see—to try and identify the correlation between certain things and your mood. And I don’t think I recognise what my triggers are.” | “for people with anxiety, there’s sometimes specific avoidance, not of everything but of certain things, and I’m just wondering how, when we think about the biological changes that occur in the body, how if they had some kind of technology on them, they would be able to reflect on the changes when they’re in the feared situations.” |  |
|  | Treatment trajectory/prevention | “if it prompted you and said, ‘It’s noticed that in the past when you’ve been less active you’ve had bad periods—And you are going through a less active period—would you like to try and do some activities to increase your mood?’ [...] I think for me that would because then it would be preventative. It would be seeing that I’m in a trajectory that’s taking me off course, it’s trying to correct that trajectory.” | “one thing that I would actually like to see would be like, I know you can do this on some smartwatches, like Maps. So like when they’re first depressed they’re kind of just staying in their house, staying in their vicinity. Same with panic disorder, they’re not going anywhere. And just that therapy just kind of seeing the radius kind of expanding would just be really fascinating because that’s kind of the goal isn’t it?” |  |
|  | Positive/negative reinforcement | “I punish myself if I don’t achieve or I haven’t done what I’m supposed to do so I don’t personally keep a record of anything.” | “you have people who- they do a lot of behavioural experiments don’t they, for their anxiety and therefore feel they aren’t making any progress, where actually, you can see that they have been confronting stressful situations regularly that might be a motivating thing for them.” |  |
|  | Rumination/health anxiety | “I don’t want to keep thinking about it and then it makes it worse, like I get stressed out and I can’t sleep [...] So I don’t want to put too much emphasis on worrying about it too much.” | “I’ve had a few patients with panic disorder who have, kind of, watch their heart rate go up which can trigger a panic attack.” |  |
| **Clinical utility** | | | | |
|  | Improve clinical experience | “Do I really have to fill this in? It just makes me like really anxious. And, I don’t know, they’re trying to like push you towards feeling worse it seems to me sometimes.” | “Some people prefer to use their electronic diaries anyway to monitor things because it’s not for everybody and there is a lot of paper in CBT.” |  |
|  | Objective measurement | — | “Some people come and say, ‘Oh, I’m feeling very very anxious.’ We don’t really have a sense of how anxious they’re feeling sometimes so I think if a watch was showing that their anxiety was a 3 over 10 compared to everyone else, that might be really useful for you to know as a therapist” |  |
|  | Outcome definition | — | “[When someone with depression improves] they appear brighter, their answers to questions become longer and more detailed. Their eyes are a little bit brighter, and the speed of responses and stuff can be quicker. But then I think if someone has GAD, they’re very much really quick to like answer and [...] with GAD it’s very like, you’re overexcited quite a lot and stuff aren’t you, so you’re very chatty” |  |
|  | Perceived effectiveness/evidence-based advice | “I think just knowing that I’m communicating with a human being, would instantly give me a lot more trust in the exercise.” | — |  |
|  | Track physical health | “I think it would be really helpful [to have a fitness tracker], particularly with people with long term health conditions” | “you’d want to capture at the time of- the time they get to sleep, how long they sleep for, when they wake up. The time of any activity, what that activity is and then try and link the heartrate information into that.” |  |

^a^cells where no direct quotes were identified for particular subthemes are left blank.
